# Biomimetic selenium nanomedicine with homologous targeting enhances hepatocellular carcinoma therapy

**DOI:** 10.1016/j.mtbio.2025.102441

**Published:** 2025-10-21

**Authors:** Fang Cui, Qi-xin Huang, Zhuo Yu, Jin-ju Yang, Shi-yu Liang, Mei-qi Wang, Jie Zhu, Chen Tian, Shao-xun Li, Hao-tian Wang, Fei Chen, Yang-jie Li, Xiaobin Feng, Rui-tian Liu, Lingxiao Zhang

**Affiliations:** a State Key Laboratory of Biopharmaceutical Preparation and Delivery, Institute of Process Engineering, Chinese Academy of Sciences, Beijing, 100190, China; b School of Medicine, Hangzhou City University, Hangzhou, 310015, China; c University of Chinese Academy of Sciences, Beijing, 100049, China; d Department of Neurology, Beijing Chaoyang Hospital, Capital Medical University, Beijing, 100020, China; e Department of Medical Oncology, Beijing Tsinghua Changgung Hospital, School of Clinical Medicine, Tsinghua Medicine, Tsinghua University, Beijing, 102218, China; f Ningxia University, Yinchuan, 750021, Ningxia, China; g Ytrrium-90 Precision Interventional Radiotherapy Center of Liver Cancer, Beijing Tsinghua Changgung Hospital, School of Clinical Medicine, Tsinghua Medicine, Tsinghua University, Beijing, 102218, China; h Interdisciplinary Nanoscience Center, Aarhus University, Aarhus C, 8000, Denmark

**Keywords:** Hepatocellular carcinoma, Biomimetic nanomedicine, Extracellular vesicles, Homotypic targeting, Selenium nanoparticles

## Abstract

Hepatocellular carcinoma (HCC) is a highly aggressive malignancy with limited effective therapeutic options. While trace elements like selenium (Se)-based nanoparticles (SeNPs) show promising anti-tumor effects, their clinical application is greatly hindered by poor targeting efficiency. Here, we engineered a biomimetic Se nanomedicine, CCEVSe, by encapsulating SeNPs within tumor cell-derived extracellular vesicles (CCEVs). Utilizing the inherent homotypic targeting and immune-evasion properties of CCEVs, CCEVSe selectively targets and is effectively internalized by tumor cells of the same origin. *In vitro* studies confirmed that CCEVSe exhibits superior cytotoxicity by amplifying intracellular reactive oxygen species and inhibiting key malignant behaviors, including proliferation, stemness, and metastasis. *In vivo* experiments in an HCC xenograft mouse model revealed that CCEVSe achieved superior tumor-specific accumulation, with Se concentrations in tumors approximately four times higher than free SeNPs after one injection. This enhanced targeting led to significant therapeutic efficacy, with CCEVSe achieving up to 87 % tumor growth suppression while exhibiting excellent safety with no observable systemic toxicity. In summary, our study provides a new paradigm for the cell-selective delivery of therapeutic nanomaterials through biomimetic modification, offering a promising strategy for precise HCC therapy.

## Graphical abstract

**Figure:**
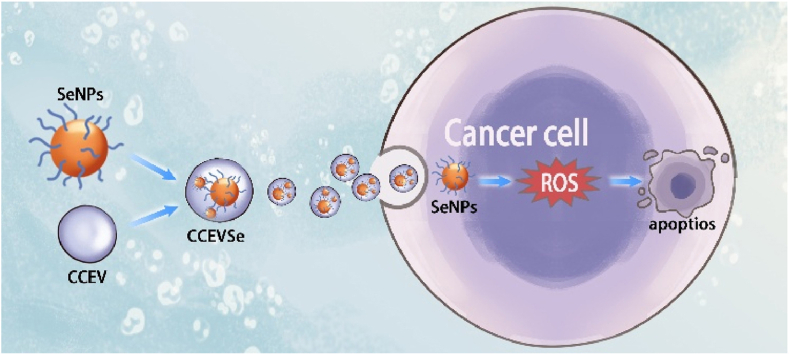

## 1 Introduction

Hepatocellular carcinoma (HCC), a major cause of liver cancer deaths, is characterized with a low 5-year survival rate [[1], [2], [3]]. Conventional targeted drugs are active in the first-line of advanced-stage HCC treatment [4,5], but face significant challenges due to drug resistance or intolerance [6,7]. Benefiting from tailored physicochemical characteristics and surface modifications, nanomedicines that specifically target and release drugs in response to endogenous or external stimuli have emerged for HCC treatment [[8], [9], [10], [11], [12]]. However, their current targeting model relies mainly on passive tumor accumulation through the enhanced permeability and retention effect [13]. A major hurdle hindering clinical translation is the uncontrolled biodistribution in normal liver tissues and other organs [14]. In contrast, biomimetic nanomedicines built from live cells, cell membranes, or extracellular vesicles have shown great potential for precise tumor targeting *in vivo* [15,16]. Among these, exosomes derived from tumor cells exhibit prolonged circulation and excellent tumor-targeting specificity due to their tumor-homologous homing effect [[17], [18], [19]].

Beyond their delivery function, the inherent physicochemical and biological properties of many nanomaterials enable them to serve as therapeutic or imaging agents [[20], [21], [22]]. Among these, selenium nanoparticles (SeNPs) have attracted particular attention for their immunological benefits and have been widely used as adjuvants to activate the immune system [23,24]. More recently, SeNPs have also been investigated as reactive oxygen species (ROS) inducers to enhance radiosensitivity or directly kill tumor cells [25,26]. Selenium itself is an essential trace element with antioxidant and detoxifying effects under physiological conditions [27]. However, in HCC, Se levels are lower than in normal liver tissue [28], yet the role of Se supplementation in cancer prevention and treatment remains controversial [29]. Emerging evidence shows that excessive Se accumulation is toxic rather than beneficial, shifting its role from antioxidant to pro-oxidant [30].Supranutritional Se induces thiol oxidation, elevates ROS, and ultimately suppresses cancer progression through caspase-3-mediated tumor cell apoptosis [28,30]. Despite these promising properties, SeNPs exhibit poor solubility, particularly in water, leading to severe aggregation and limited tumor penetration [31]. Thus, improving SeNP solubility, reducing nonspecific accumulation in normal liver tissues, and preventing clearance by the hepatic reticuloendothelial system remain urgent challenges.

**Fig. 1:**
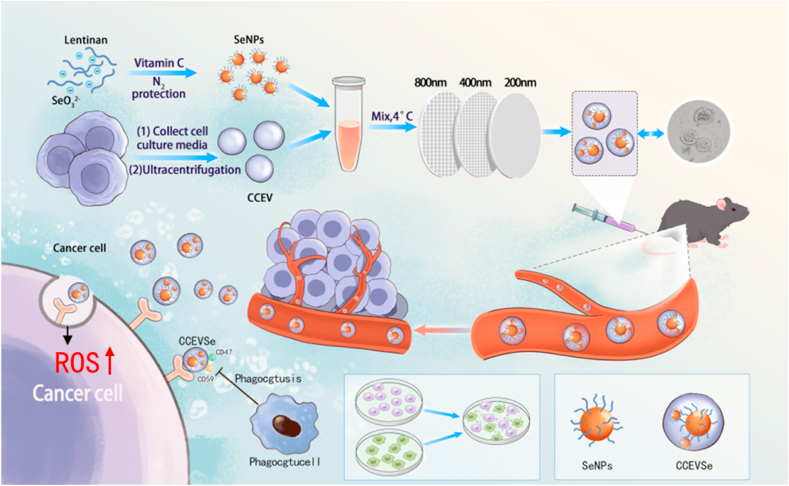
Schematic illustration of the preparation and application of CCEVSe nanomedicine for precise HCC therapy. Sodium selenite and lentinan were mixed under nitrogen protection and reduced by vitamin C to form SeNPs, which were then co-extruded with CCEVs to generate CCEVSe. CCEVSe inherits key properties of CCEVs, including homotypic tumor targeting and macrophage evasion. After reaching tumor tissue, CCEVSe delivers SeNPs efficiently into homologous tumor cells, where SeNPs are released to elevate ROS levels and trigger tumor cell death.

In this study, we constructed a biomimetic Se nanomedicine (CCEVSe) for selective targeting and killing of HCC in mice by utilizing SeNPs as ROS inducers (Fig. 1). To improve solubility, SeNPs were first stabilized with lentinan, then encapsulated into HepG2 cell line–derived cancer cell extracellular vesicles (CCEVs). Leveraging the homotypic targeting and immune evasion capabilities of CCEVs [[32], [33], [34]], CCEVSe can effectively escape macrophage clearance in the liver and precisely localize to HCC cells. Once internalized, SeNPs elevate intracellular ROS stress and promote tumor cell death, thereby effectively inhibiting HCC progression in mice. This work presents a novel strategy for precise HCC therapy that integrates tumor-derived CCEVs with an essential nutritional element, offering promising potential for clinical translation.

## 2 Materials and methods

### 2.1 Reagents and antibodies

Anti-histone H3 Antibody (Beyotime, AF0009), syntenin-1 antibody (Santa Cruz, sc-515538), anti-CD63 antibody (Solarbio, K007602P), MTT (Beyotime, ST316), Crystal violet (Beyotime, Y268091-100g), anti-CD44 antibody (Solarbio, K015936M), anti-CD44 antibody (MCE, HY-P80054), low attachment cell culture plates (Biofil, TCP030006), Annexin V-FITC Apoptosis Detection Kit (Beyotime, C1062S).

### 2.2 Preparation of SeNPs

Under a nitrogen-protected environment, 60 mg of lentinan (Solarbio, L8270-10g) and 55 mg (0.318 mmol) of sodium selenite (MCE, HY-W686381) were added to a round-bottom flask, followed by 45 mL of double-distilled water. The mixture was magnetically stirred for 20 min. Then, 352 mg (2 mmol) of vitamin C powder (Aladdin, A103535-100g) was dissolved in 5 mL of double-distilled water, and the vitamin C solution slowly added dropwise into the flask using a disposable syringe. The reaction was allowed to proceed for 24 h. After completion, the mixture was filtered through a 0.22 μm membrane and dialyzed in an activated 14,000 D dialysis bag (Beyotime, FDM714-5m), with the external water replaced every 3 h for 24 h. The final selenium concentration was determined by ICP-MS. The particle size and morphology of the nanoparticles were characterized using dynamic light scattering (DLS) and transmission electron microscopy (TEM).

### 2.3 Preparation of green fluorescent-labeled SeNPs

Under nitrogen protection, 60 mg of lentinan, 55 mg (0.318 mmol) of sodium selenite, and 5 mg of 7-amino-4-(trifluoromethyl) coumarin (AFC) (Macklin, A801693-250 mg) were added to a round-bottom flask with 45 mL of double-distilled water, and the mixture was stirred for 20 min. Then, 352 mg (2 mmol) of vitamin C powder dissolved in 5 mL of double-distilled water was added dropwise into the flask via a syringe, and the reaction was maintained for 24 h. Subsequent steps (filtration, dialysis, and characterization) were identical to those for unlabeled SeNPs.

### 2.4 Construction of plasmids to enhance EV secretion

Based on literature reports, a plasmid expressing three genes (Steap3, syndecan-4, and a fragment of L-aspartate oxidase, NadB214-514) was constructed using PcDNA3.1, named PcDNA3.1-CMV-STEAP3-IRES-syndecan-4-P2A-NadB214-514 (Pc3.1-SSN). This plasmid significantly enhances EV secretion, as reported in the literature. A schematic of the plasmid is shown below.

### 2.5 Preparation of natural tumor-derived extracellular vesicles

5 × 10^6^ HepG2 cells (obtained from the Procell) were seeded in 10 mL of DMEM complete medium containing 10 % FBS (Huanke, HK-U500) and 1 % penicillin-streptomycin. When the cell confluency reached 80–90 %, the medium was replaced with serum-free DMEM, and the cells were cultured for 48 h. The EV-containing medium was collected and subjected to sequential centrifugation: 600×*g* for 30 min (to remove cells) and 2000×*g* for 30 min (to remove apoptotic bodies). EVs were precipitated by ultracentrifugation at 130,000×*g* for 80 min using an Optima XPN-100/XPN-90 ultracentrifuge with a Type 45 Ti rotor, then resuspended in PBS.

### 2.6 Preparation of cancer cell-derived extracellular vesicles (CCEV)

5 × 10^6^ HepG2 cells were seeded in 10 mL of DMEM complete medium containing 10 % FBS and 1 % penicillin-streptomycin. At 80–90 % confluency, the Pc3.1-SSN plasmid was transfected into HepG2 cells using PEI at the recommended PEI-DNA ratio. After 6 h of transfection, the medium was replaced, and the cells were cultured for another 36–48 h. The EV-containing medium was collected and subjected to sequential centrifugation: 600×*g* for 30 min (to remove cells) and 2000×*g* for 30 min (to remove apoptotic bodies). EVs were precipitated by ultracentrifugation at 130,000×*g* for 80 min using an Optima XPN-100/XPN-90 ultracentrifuge with a Type 45 Ti rotor, then resuspended in PBS.

### 2.7 Preparation of CCEV-SeNPs

1 mL of collected CCEV suspension (at a protein concentration of 0.5 mg/mL) was mixed with 1 mL of lentinan-SeNPs solution. The mixture was extruded through a liposome extruder (Avanti, 610000) using 0.8 μm, 0.4 μm, and 0.2 μm polycarbonate nucleopore membranes (Whatman) sequentially. The final solution was collected, and selenium content was determined by ICP-MS. Particle size was characterized by DLS and TEM.

### 2.8 Preparation for AFC-labeled CCEV-SeNPs

The procedure was identical to that for unlabeled CCEV-SeNPs, except that AFC-labeled SeNPs were used instead of unlabeled SeNPs.

### 2.9 Transmission electron microscopy (TEM)

Take 50 μL of CCEV-SeNPs or SeNPs suspension and drop it onto the smooth surface of a parafilm. Place a copper grid over the droplet and let it stand for 20 min. Blot excess liquid from the copper grid using filter paper, rinse with PBS three times, fix with 1 % glutaraldehyde solution for 5 min, and rinse with double-distilled water five times. Stain negatively with phosphomolybdic acid for 2 min, blot excess liquid, and dry at room temperature for 24 h before observation under a transmission electron microscope.

### 2.10 Preparation of HepG2-mcherry stable cell line

HepG2 cells were incubated with a control Lentivirus carrying mCherry and puromycin resistance (purchased from Obio Technology). The medium was replaced every 24 h. After 72 h, complete medium containing 2 μg/mL puromycin was added. During cell expansion and passage, the puromycin concentration was gradually increased to 8 μg/mL.

### 2.11 MTT assay

Prepare cell suspension in the logarithmic growth phase using the same method as cell passaging. Dilute the suspension with complete medium to a final density of 20,000 cells/mL (total volume 10 mL). Pipette 100 μL of the cell suspension into each well of a 96-well plate and incubate in a cell culture incubator for 12–24 h until cells adhere and reach 20–30 % confluency. After drug administration, return the plate to the incubator. When the control group reaches 80–90 % confluency, add 20 μL of 5 × MTT solution to each well and incubate at 37 °C for 4 h. Add 100 μL of DMSO to each well, gently shake the plate to fully dissolve formazan crystals, and measure absorbance at 570 nm using a microplate reader [35].

### 2.12 Monoclonal colony formation assay

Dilute HepG2 cell suspension with complete medium to a final density of 3000 cells/mL. Add 3 mL of complete medium to each well of a 6-well plate, followed by 1 mL of the diluted cell suspension. Incubate in a constant-temperature cell incubator for 24 h, then add SeNPs or CCEV-SeNPs at different concentrations. Incubate for another 6 days. Remove the medium, rinse with PBS three times, stain with crystal violet for 20 min, remove the stain, and rinse with PBS three times. Count the number and measure the size of clonal colonies per well.

### 2.13 Tumor spheroid formation assay

Prepare HepG2 cell suspension in the logarithmic growth phase and dilute with stem cell medium (10 % FBS, DMEM/F12, 1 % penicillin-streptomycin, B27 (Yeasen, 60703ES03), 20 ng/mL bFGF (Yeasen, 91330ES10), 20 ng/mL EGF (Yeasen, 92708ES60)) to a final density of 3000 cells/mL (total volume 18 mL). Add 6 mL of the cell suspension to each well of a low-attachment 6-well plate. After 4–5 days, small cell clusters will form. Centrifuge at 200×*g* for 5 min, replace 50 μL of medium, add SeNPs or CCEV-SeNPs, and continue culturing with 50 μL medium replacement every 4–5 days. After 12 days, observe the inhibitory effect of the nanoparticles on tumor spheroid formation.

### 2.14 Cell scratch assay

HepG2 cells in the logarithmic growth phase were digested into single-cell suspension and seeded in 24-well plates. After 24 h of culture at 37 °C with 5 % CO_2_, scratches were made vertically using a 200 μL pipette tip. Cells were rinsed three times with PBS to remove detached cells, then incubated with low-serum medium (2 % FBS in DMEM) containing SeNPs or CCEV-SeNPs. Images were taken after 48 h, and the average scratch distance was calculated using ImageJ software.

### 2.15 Cell apoptosis detection

Following the instructions of the Annexin V-FITC Apoptosis Detection Kit (Beyotime, C1062L): Transfer the medium from the culture dish to a clean EP tube, centrifuge at 1000×*g* for 5 min to collect floating cells, and resuspend in PBS. Rinse adherent cells once with PBS, digest with EDTA-free trypsin (EDTA may interfere with Annexin V-FITC binding to phosphatidylserine), add 20 μL serum, and centrifuge at 800 rpm for 5 min. Combine with the suspended floating cells, take 1 × 10^6^ cells, centrifuge at 1000×*g* for 5 min, remove the supernatant, and gently resuspend in 195 μL Annexin V-FITC binding buffer. Add 5 μL Annexin V-FITC and 10 μL propidium iodide staining solution, mix gently, incubate at room temperature in the dark for 10–20 min (resuspend 2–3 times during incubation to improve staining), place on ice, and detect using flow cytometry [36,37].

### 2.16 Induction of THP-1 cells into macrophages

THP-1 cells were cultured in RPMI 1640 medium containing 10 % FBS, 2 μM L-glutamine, 1 % penicillin-streptomycin, and 0.05 μM 2-mercaptoethanol for expansion. THP-1 cells were seeded in plates at a density of 5 × 10^5^ cells/mL and treated with PMA (100 ng/mL) for 24 h to induce differentiation.

### 2.17 Cell uptake assay

Logarithmic growth phases cells were digested. 293T and THP-1 cells were stained with Hoechst 33342, rinsed three times with PBS, and seeded on PDL-coated coverslips (Beyotime, 1070000). HepG2-mcherry cells were directly seeded on coverslips. After complete adherence, fluorescently labeled SeNPs or CCEV-SeNPs were added. Cellular uptake was observed, and fluorescence intensity was analyzed using ImageJ.

### 2.18 Cell co-culture assay

HepG2-mcherry cells were co-cultured with 293T or THP-1 cells at a 1:1 ratio. After complete adherence, fluorescently labeled SeNPs or CCEV-SeNPs were added. Under fluorescence microscopy, HepG2-mcherry cells appeared red, while 293T or THP-1 cells only showed blue nuclei (no visible cell morphology). Since nanoparticles were labeled with AFC, the location and intensity of green fluorescence indicated the number of nanoparticles taken up by cells.

### 2.19 Determination of mitochondrial membrane potential

The enhanced mitochondrial membrane potential assay kit containing JC-1 (Beyotime, C2003S) was used [38,39]. JC-1 was diluted by adding 5 μL of JC-1 (200 × ) to 1 mL of JC-1 staining buffer. Cells were seeded in confocal culture dishes. After drug treatment, the culture dishes were taken out and washed with PBS. Next, 1 mL of cell culture medium and 1 mL of JC-1 staining solution were added, and the mixture was incubated at 37 °C for 20 min. Then, the supernatant was removed, and the cells were washed with JC-1 staining buffer. Finally, 2 mL of cell culture medium was added, and the cells were observed under a laser confocal microscope. The excitation wavelength was set to 490 nm and the emission wavelength to 530 nm to detect JC-1 monomers. The excitation wavelength was set to 525 nm and the emission wavelength to 590 nm to detect JC-1 aggregates.

### 2.20 Establishment of HepG2 xenograft tumor model in Balb/C nude mice

HepG2 cells in the logarithmic growth phase were digested, centrifuged, and rinsed three times with PBS. Resuspend cells in physiological saline or PBS to a density of 2–3 × 10^7^ cells/mL and keep on ice. Thirty 6–8-week-old female Balb/C nude mice (18–20 g) were subcutaneously injected with 100 μL of cell suspension into the right leg. After 2 weeks, successful tumor formation was confirmed. When tumors grew to ∼100 mm^3^, mice were divided into 5 groups (6 mice/group) for treatment: model control, low-dose SeNPs, high-dose SeNPs, low-dose CCEV-SeNPs, and high-dose CCEV-SeNPs. Drugs were administered via tail vein injection every 3 days, with doses based on selenium content detected by ICP-MS: control (saline), low-dose SeNPs (12.5 μM, 200 μL), high-dose SeNPs (25 μM, 200 μL), low-dose CCEV-SeNPs (12.5 μM, 200 μL), and high-dose CCEV-SeNPs (25 μM, 200 μL). After treatment, equal weights of heart, liver, spleen, lung, kidney, and tumor tissues were collected for selenium content detection by ICP-MS. Remaining tissues were fixed in 4 % paraformaldehyde for pathological analysis. All animal experiments were performed in accordance with the China Public Health Service Guide for the Care and Use of Laboratory Animals. Experiments involving mice and protocols were approved by the Institutional Animal Care and Use Committee of Institute of Process Engineering, Chinese Academy of Sciences (IPEAECA2022068).

### 2.21 Immunohistochemistry and hematoxylin and eosin (H&E) staining of tumor tissues

After treatment, Balb/C nude mice were euthanized by cervical dislocation. Tumor tissues were separated, rinsed in PBS, freed of connective tissue, and fixed in 4 % paraformaldehyde for 48 h. After rinsing with pure water, the largest section was trimmed with a scalpel. Tissues were dehydrated, embedded in paraffin, and sectioned. Sections were baked, dewaxed, rehydrated, subjected to antigen retrieval, hydrogen peroxide depletion, blocking, antibody incubation, DAB staining, dehydration, and mounting. HE staining was performed according to the kit instructions (Solarbio, G1120).

### 2.22 Statistical analysis

Data are presented as the mean ± standard error of the mean (SEM) based on at least triplicate experiments. The means were analyzed by one-way or two-way analysis of variance as indicated, followed by multiple comparisons using Tukey's test within the Prism software package (Prism 10.2.1; GraphPad Software, USA, 2024).

## 3 Results

### 3.1 Characterization of biomimetic CCEVSe

**Fig. 2:**
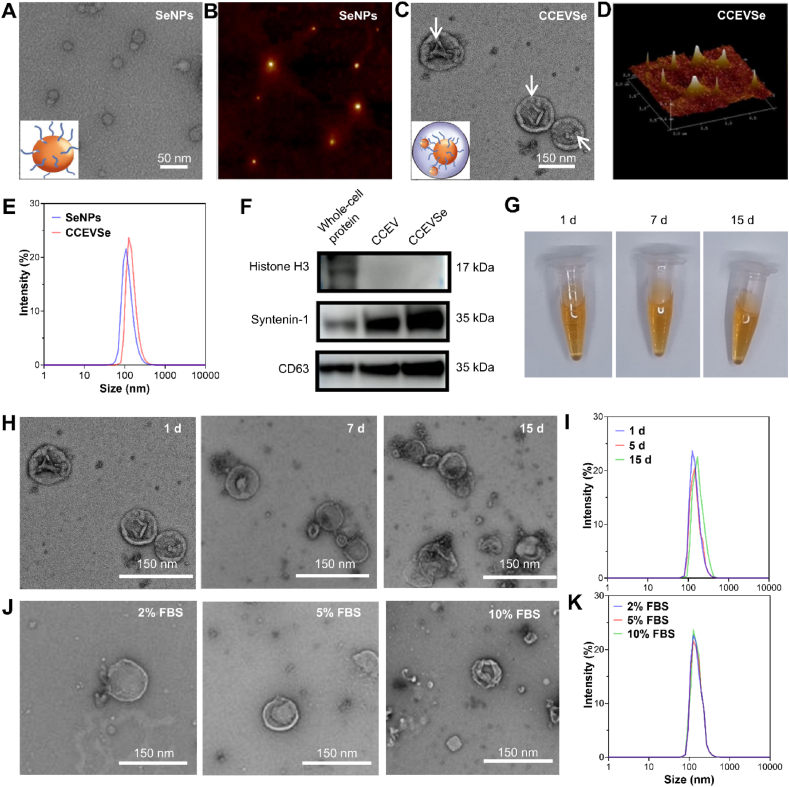
Characterization of biomimetic CCEVSe. (A–D) TEM and AFM images of SeNPs and CCEVSe. (E) DLS analysis of SeNPs and CCEVSe. (F) Western blot detection of extracellular vesicle markers. (G) Appearance of SeNPs and CCEVSe after storage at 4 °C for different durations. (H) TEM images of CCEVSe after different days storage at 4 °C. (I) DLS results of CCEVSe after different days storage at 4 °C. (J) TEM Images of CCEVSe after 5-day storage at 4 °C with different serum contents. (K) DLS results of CCEV-SeNPs after 5-day storage at 4 °C with different serum contents.

TEM images reveal the successful synthesis of lentinan-conjugated SeNPs with an average particle size of ∼25 nm (Fig. 2A). The particle size was further validated by atomic force microscopy (AFM) (Fig. 2B). Encapsulation of SeNPs within CCEVs was also confirmed *via* TEM, which showed the SeNPs to be uniformly distributed within the CCEV lumen (Fig. 2C). The particle size of CCEVSe, as determined by AFM, was approximately ∼170 nm, which was comparable to the ∼145 nm hydrodynamic diameter measured by dynamic light scattering (DLS; Fig. 2D and E). Notably, the DLS-measured size of bare SeNPs was significantly larger than that observed by TEM, primarily due to the presence of surface polysaccharides that increased the hydrodynamic diameter. After encapsulation with SeNPs (−52 mV), the surface charge of CCEVSe decreased from −15 mV of CCEVs to −25 mV), indicating that SeNPs were successfully loaded into the inner cavity of the CCEVs (Table S1). Western blot analysis detected exosome markers, including syntenin-1, CD63, and histone H3, in both CCEVs and CCEVSe. Importantly, the SeNP encapsulation did not compromise marker expression, indicating that CCEVSe retained the biological characteristics of native CCEVs (Fig. 2F). Inductively coupled plasma mass spectrometry (ICP-MS) further confirmed selenium incorporation, with a quantified concentration of 35.27 ± 0.35 mg L^−1^ (141 μg Se per mg protein, Table S1). For subsequent experiments, drug concentrations were normalized to the selenium content. The stability of CCEVSe was then assessed. Samples stored at 4 °C showed no visible changes in appearance over time (Fig. 2G). TEM and DLS analyses confirmed good dispersibility and a stable size distribution in PBS after storage for up to 15 days (Fig. 2H and I). Furthermore, particle integrity was unaffected by varying concentrations of fetal bovine serum (FBS; Fig. 2J and K). Collectively, these results demonstrate the successful synthesis of a homogeneous and stable CCEVSe suspension.

### 3.2 CCEVSe homotypically targets HepG2 tumor cells

**Fig. 3:**
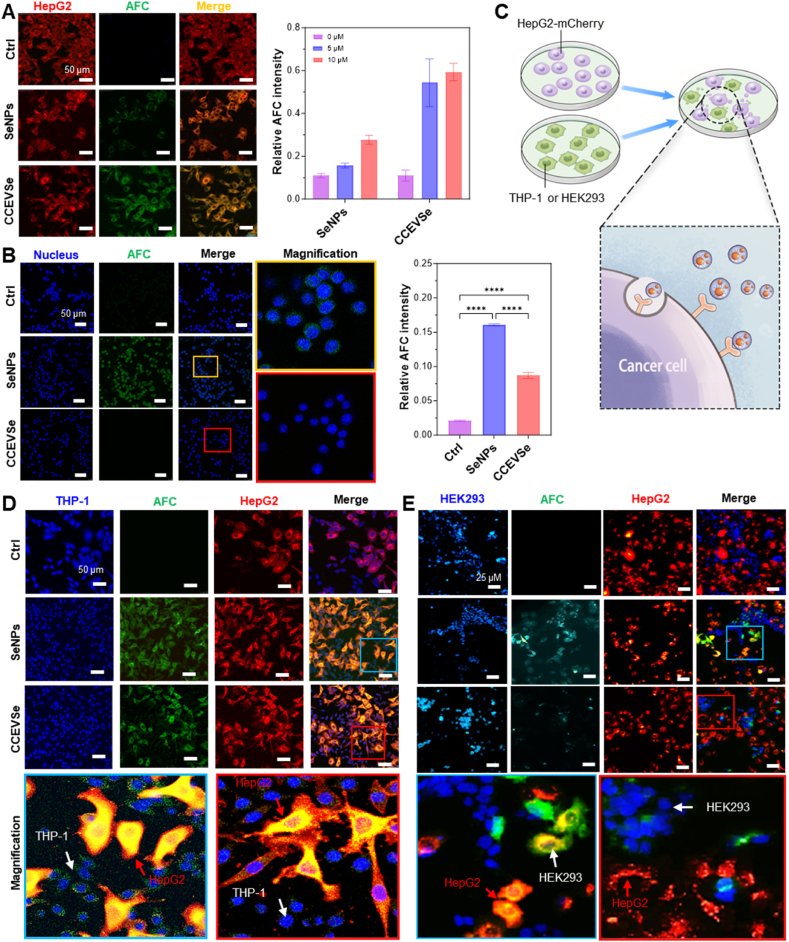
Targeting and specificity of CCEVSe to tumor cells. (A,B) Cellular uptake of CCEVSe versus free SeNPs by HepG2-mCherry cells (A) and THP-1 differentiated macrophages (B). (C) Schematic illustration of the co-culture system designed to evaluate the targeting specificity of CCEVSe and free SeNPs. (D,E) Analysis of cellular uptake in a co-culture system of HepG2-mCherry cells and THP-1 differentiated macrophages (D) or HEK293 cells (E). Data are presented as the mean ± SEM. The *p* values were analyzed by one-way analysis of variance, followed by multiple comparisons using Tukey's test. ∗*p* < 0.05, ∗∗*p* < 0.01, ∗∗∗*p* < 0.001, ∗∗∗∗*p* < 0.0001.

To evaluate the homotypic targeting advantage conferred by HepG2-derived CCEVs, we investigated the cellular uptake of CCEVSe, prepared with AFC-labeled SeNPs (green fluorescence), in HepG2-mCherry cells (red fluorescence). As demonstrated by fluorescence imaging, CCEVSe was internalized with markedly greater efficiency by HepG2-mCherry cells compared to free SeNPs, as evidenced by a significantly stronger green fluorescence signal (Fig. 3A). The CCEVSe also exhibited the macrophage-evasion property characteristic of CCEVs. The uptake of CCEVSe by THP-1-differentiated macrophages was significantly reduced; SeNPs were readily internalized by these cells, whereas CCEVSe uptake was minimal, with the green fluorescence intensity in CCEVSe-treated cells being approximately half that in SeNP-treated cells (Fig. 3B). Similarly, when co-cultured with irrelevant human embryonic kidney HEK293 cells, CCEVSe uptake was minimal, while the SeNPs were readily internalized (Fig. S1). Meanwhile, quantitative analysis of the cellular uptake of SeNPs and CCEVSe by HepG2-mCherry, HEK293, and THP-1 cells further confirmed the targeting specificity of CCEVSe (Fig. S2). These collective results indicate that CCEVSe can specifically recognize and target cells of the same origin, thereby circumventing immune clearance and reducing non-specific cellular uptake.

To further verify whether CCEVSe can specifically target HepG2 in a more complex cellular environment, we constructed a competitive co-culture system containing HepG2-mCherry cells (red fluorescence) alongside either THP-1 differentiated macrophages or HEK293 cells (stained with Hoechst 33342, blue fluorescence) (Fig. 3C). In the HepG2-mCherry/THP-1 co-culture system, free SeNPs were rapidly internalized by both cell types, resulting in visible green fluorescence in THP-1 cells and merged yellow fluorescence in HepG2-mCherry cells, indicating a widespread and non-specific distribution. In sharp contrast, CCEVSe was almost exclusively co-localized with HepG2-mCherry cells, as evidenced by the yellow fluorescence being confined solely to the red-fluorescent cells, while no discernible green fluorescence was observed in the blue-fluorescent THP-1 cells (Fig. 3D). This is largely attributed to the expression of CD47 and CD59 in CDEV (Fig. S3), which effectively reduced macrophage uptake [40,41]. A similar homotypic targeting phenomenon was also observed in the HepG2-mCherry/HEK293 co-culture system, where CCEVSe uptake was restricted to the HepG2-mCherry cells (Fig. 3E). These results conclusively demonstrate the specific targeting capability of CCEVSe in a competitive and biologically relevant setting.

### 3.3 CCEVSe specifically inhibits HepG2 tumor cell growth

**Fig. 4:**
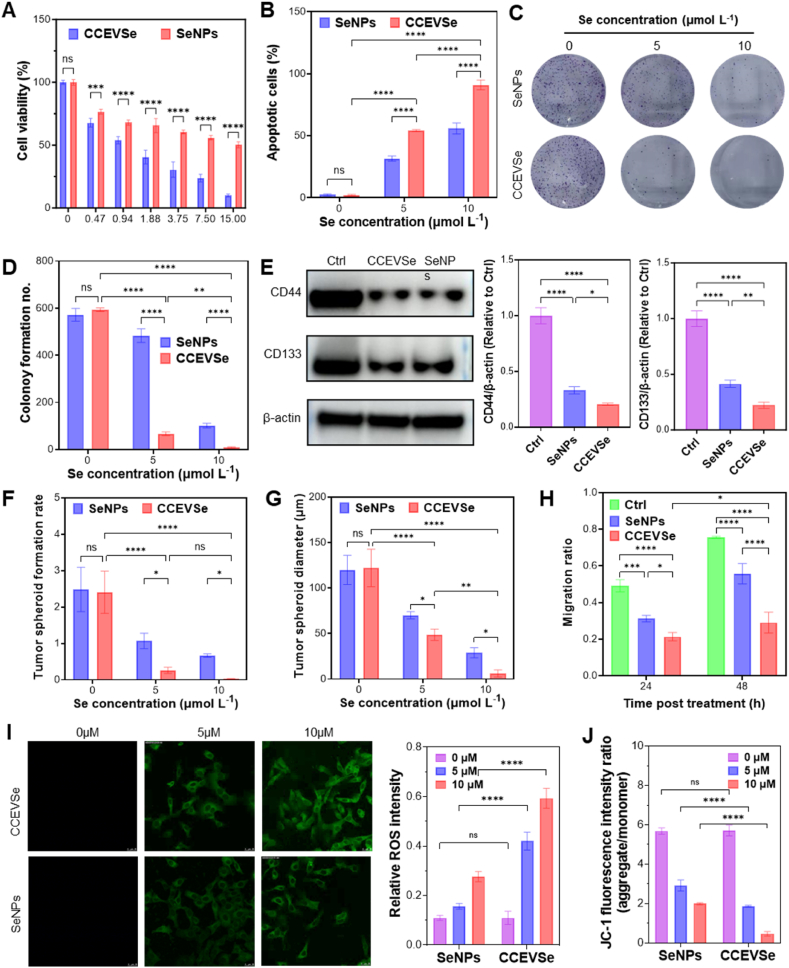
Evaluation of the cytotoxic, anti-proliferative, and inhibitory effects of CCEVSe on HepG2 tumor cells. (A) Dose-dependent killing effects of CCEVSe and SeNPs on HepG2 cells measured by MTT assay. Cells were treated with concentrations of 0, 0.47, 0.9375, 1.88, 3.75, 7.5, and 15 μM in complete medium for 48 h (n = 3 per concentration). (B) Quantification of apoptotic cells by flow cytometry. (C) Inhibition of colony formation by CCEVSe and SeNPs observed in plate colony formation assay. (D) Statistical analysis corresponding to panel C. (E) Western blot analysis of tumor stem cell markers CD44 and CD133 after treatment with CCEVSe or SeNPs in ultra-low adhesion plates with stem cell medium and growth factors. (F,G) Quantitative analysis of tumor spheroid formation at different time points after treatment with CCEVSe and SeNPs in ultra-low adhesion culture. (H) Inhibition of HepG2 cell migration detected by scratch assay. (I) Detection of intracellular ROS by ROS probe. (J) Analysis of mitochondrial membrane potential using the JC-1 fluorescent probe. Data are presented as the mean ± SEM. The *p* values were analyzed by one-way analysis of variance, followed by multiple comparisons using Tukey's test. ∗*p* < 0.05, ∗∗*p* < 0.01, ∗∗∗*p* < 0.001, ∗∗∗∗*p* < 0.0001.

As shown in Fig. S4, CCEV itself showed almost no cytoxicity to HepG2 cells, and the cytotoxicity of simple mixture of CCEV and SeNPs (CCEV&SeNPs) was similar to SeNPs. In sharp contrast, CCEVse with enhanced cellular delivery functions exhibited much higher cytotoxicity, this is largely attributed to the enhanced internalization of CCEVSe by HepG2 cells. Compared to free SeNPs, CCEVSe exhibited significantly higher cytotoxicity toward HepG2 cells at the same selenium concentration, with a lower IC_50_ of 1.207 μM (Fig. 4A). Cytotoxicity analysis further demonstrated that CCEVSe was more potent against HepG2 cells than the first-line therapeutic agents Sorafenib and Doxorubicin (Fig. S5). Apoptosis analysis confirmed this effect, showing that approximately 55 % of HepG2 cells underwent apoptosis in the CCEVSe-treated group, compared to 35 % in the SeNP-treated group, at a Se concentration of 5 μmol L^−1^. When the concentration was increased, the apoptotic percentages further rose to 94 % and 56 % for CCEVSe and SeNPs, respectively (Fig. 4B). In other hepatocellular carcinoma (HCC) cell lines, such as PLC and Hep-1, CCEVSe prepared by encapsulating SeNPs into the corresponding cell-line-derived CCEVs also showed significantly higher cytotoxicity than the free SeNPs (Fig. S6). To further verify the homologous targeting specificity, CCEVSe prepared from Hep-1 cell–derived CCEV was added to HepG2 cells. The cytotoxicity of Hep-1 CCEVSe toward HepG2 cells was even lower than that of SeNPs (Fig. S7A). Conversely, HepG2-derived CCEVSe exhibited limited cytotoxicity toward Hep-1 cells (Fig. S7B). These results collectively demonstrate that the cell-selective uptake of CCEVSe enables effective killing of corresponding tumor cells while maintaining improved biosafety for off-target cells. Interestingly, when free SeNPs and HepG2-derived CCEVSe were applied to non-target THP-1 and HEK293 cells, the non-specific uptake of SeNPs by these cells caused significant cell death. In contrast, minimal cell death was observed in cells treated with CCEVSe due to its reduced uptake (Fig. S8).

Subsequently, the impacts of CCEVSe on tumor cell proliferation, stemness, and metastasis were further evaluated. First, CCEVSe effectively inhibited the proliferation of tumor cells in a concentration-dependent manner. Compared to free SeNPs, the number of tumor clonal colonies formed in the CCEVSe-treated group was only approximately one-seventh of that observed in the SeNP-treated group at a selenium concentration of 5 μmol L^−1^. At a Se concentration elevated to 10 μmol L^−1^, tumor clonal colonies were almost entirely suppressed in the CCEVSe-treated group, while distinct colonies were still observed in the SeNP-treated group (Fig. 4C and D). Moreover, tumor stemness was assessed by analyzing the expression of stemness markers, including CD44 and CD133. Although SeNPs also significantly reduced the expression of these markers in HepG2 cells, the levels of CD44 and CD133 were even lower in the CCEVSe-treated group (Fig. 4E). Tumor stemness was also reflected by spheroid formation, as the ability of HepG2 cells to form spherical aggregates in suspension is a key indicator of their stem-like properties. Microscopic observation revealed that CCEVSe treatment dramatically inhibited tumor spheroid formation compared to SeNPs at the same selenium concentration, which was reflected in a lower spheroid formation rate and a smaller diameter of the formed spheroids (Fig. 4F, G, Fig. S9). Furthermore, the capacity for tumor metastasis was evaluated using a scratch assay. CCEVSe also significantly inhibited tumor cell migration, as the gap between the CCEVSe group and the SeNPs or control groups widened considerably over time (Fig. 4H, Fig. S10).

As a potent inducer of ROS, SeNPs are known to increase intracellular ROS levels in a concentration-dependent manner to trigger tumor cell death [42,43]. Our results demonstrate that the enhanced intracellular delivery of SeNPs by CCEVSe also resulted in a significantly higher level of ROS in HepG2 cells (Fig. 4I). This was further supported by mitochondrial membrane potential analysis, which showed that CCEVSe induced a more significant loss of mitochondrial membrane potential in HepG2 cells compared to SeNPs, thereby confirming that CCEVSe mediates tumor cell death *via* a ROS-dependent pathway (Fig. 4J). Collectively, these findings indicate that the enhanced tumor-targeted delivery achieved by CCEVs amplified the intrinsic cytotoxicity of SeNPs by promoting tumor cell apoptosis and necrosis, while effectively inhibiting tumor cell proliferation, stemness, and metastasis.

### 3.4 CCEVSe effectively inhibits HepG2 tumor progression

**Fig. 5:**
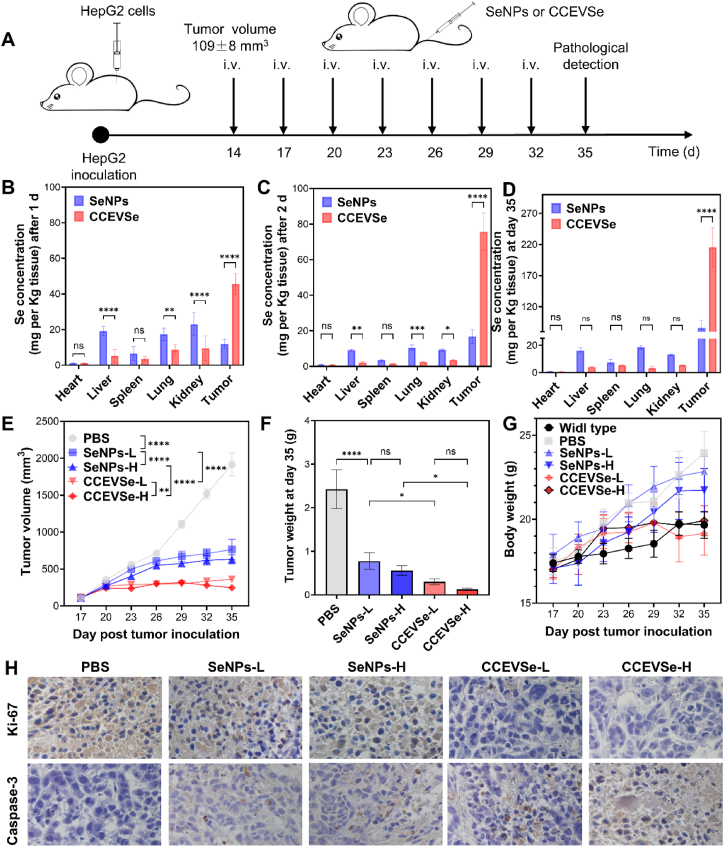
Therapeutic effects of CCEVSe in HepG2 tumor xenograft mouse models. (A) Schematic of the treatment regimen. (B,C) Selenium content in major organs at 24 h and 48 h after a single tail vein injection of SeNPs or CCEVSe. (D) Selenium content in tissues detected by ICP-MS at the end of treatment. (E) Tumor volume changes in xenograft mice following intravenous injections of CCEVSe or SeNPs at different doses. (F) Weight of isolated tumor tissues at the end of treatment. (G) Changes in mouse body weight during treatment. (H) Immunohistochemical analysis of Ki-67 and caspase-3 expression in tumor tissues. Data are presented as the mean ± SEM. The *p* values were analyzed by one-way analysis of variance, followed by multiple comparisons using Tukey's test. ∗*p* < 0.05, ∗∗*p* < 0.01, ∗∗∗*p* < 0.001, ∗∗∗∗*p* < 0.0001.

The in vivo therapeutic efficacy of CCEVSe was evaluated in a subcutaneous HCC xenograft mouse model established by injecting HepG2 cells into nude mice and treated with SeNPs or CDEVSe (Fig. 5A and Table S2). The CCEVSe-treated group exhibited excellent therapeutic efficacy, leading to a significant prolongation of survival time for tumor-bearing mice (Fig. S11A). The biodistribution of CCEVSe in major organs was investigated following a single intravenous (i.v.) injection by analyzing selenium concentration *via* ICP-MS. As shown in Fig. 5B, the tumor demonstrated the highest selenium concentration at approximately 45.7 mg Se per g of tumor tissue after 1 day, a concentration approximately four times greater than that achieved with free SeNPs. In contrast to free SeNPs, which exhibited high accumulation in the liver, lung, and kidney, significantly less CCEVSe accumulated in these organs, which is largely attributed to the macrophage and renal cell evasion properties conferred by CCEVs (Fig. 3D–E). When the time was extended to 2 days post-injection, the selenium concentration in tumors further increased to 75.7 mg Se per g of tumor tissue, approximately 1.7 times the concentration detected at day 1. In contrast, the selenium levels in the tumors and other organs of SeNP-treated mice increased only slightly (Fig. 5C). These data provide strong evidence that the encapsulation of SeNPs within CCEVs substantially increases their circulation time and tumor-targeting ability.

Subsequently, all HCC mice received seven injections of the indicated formulations at 3-day intervals, beginning on day 14 (Fig. 5A). At the end of the experiment on day 35, the Se concentration in tumor tissues reached approximately 215.7 mg Se per g, which was about 2.5 times that in the SeNP-treated group. Meanwhile, the selenium concentrations in the liver, lung, and kidney remained similar to those observed after a single injection (Fig. 5D), indicating that CCEVSe specifically accumulates in the tumor without causing marked selenium accumulation in metabolic organs. As expected, HCC mice treated with low- or high-dosage CCEVSe achieved comparably high tumor suppression efficiencies of 81 % and 87 %, respectively, at day 35. In contrast, treatment with SeNPs at the corresponding selenium concentrations only yielded tumor suppression efficiencies of 60 % for the SeNPs-L group and 67 % for the SeNPs-H group (Fig. 5E). Consistent with these findings, the tumor weights detected on day 35 showed a similar trend (Fig. 5F). Notably, the body weight of the mice was not significantly affected by the injection of SeNPs or CCEVSe at either dosage (Fig. 5G). Furthermore, H&E staining of major organs (heart, liver, spleen, lung, and kidney) collected from CCEVSe-H-treated mice showed no obvious histopathological damage (Fig. S11B). In contrast, a remarkable inhibition of proliferation and promotion of apoptosis were observed in tumor tissues, as evidenced by decreased Ki67 expression and increased Caspase-3 levels, respectively (Fig. 5H). While SeNP treatment increased levels of pro-inflammatory cytokines, including TNF-α and IFN-γ, this effect was effectively mitigated by CCEVSe through reduced off-target interactions (Fig. S12). Furthermore, CCEVSe at a high Se concentration of 35 μmol mL^−1^ caused only a very low hemolysis percentage of ∼0.15 % (Fig. S13), well below the 0.8 % threshold considered risky. Therefore, these results collectively indicate that CCEVSe specifically enriches SeNPs in tumor tissues to inhibit proliferation and promote apoptosis, all while exhibiting negligible systemic side effects.

## 4 Discussion

In this study, we successfully engineered a biomimetic nanomedicine, CCEVSe, by encapsulating SeNPs within HepG2-derived CCEVs. By exploiting the inherent homotypic targeting and immune-evasion capacities of CCEVs, CCEVSe achieved potent therapeutic efficacy both *in vitro* and *in vivo* (Fig. 4, Fig. 5). Importantly, CCEVSe selectively accumulated in HepG2 tumor cells, where it markedly enhanced intracellular ROS levels (Fig. 3). This effect translated into the inhibition of key malignant behaviors such as proliferation, stemness, and metastasis, while avoiding significant off-target toxicity (Fig. 4). Furthermore, in xenograft models, CCEVSe treatment effectively suppressed tumor growth without causing abnormal selenium accumulation in healthy tissues or systemic toxicity (Fig. 5).

The therapeutic benefits of CCEVSe stem from several unique advantages. First, the use of endogenous, tumor-derived CCEVs enables highly specific drug delivery, thereby reducing systemic side effects. Second, the homotypic recognition of CCEVs offers a promising strategy to overcome intratumoral heterogeneity, a major limitation in conventional nanomedicine delivery. Third, the intrinsic ability of CCEVs to evade clearance by macrophages and renal filtration prolongs circulation, allowing for improved tumor accumulation. Thus these features address critical barriers of current nanomedicine platforms, particularly poor selectivity and organ-related toxicity.

Our findings also highlight the translational potential of CCEV-based biomimetic nanomedicines. Beyond SeNPs, the CCEV platform may be broadly applicable for delivering diverse therapeutic payloads, providing a versatile strategy for precision oncology. However, the clinical translation of this approach is not without challenges. Theoretically, personalized CCEVSe can be prepared using patient-derived primary tumor cells; however, issues such as the culture efficiency of primary cells, large-scale production of CCEVs, and long-term safety remain to be resolved. Currently, this approach is only in the exploratory stage of laboratory research.

In conclusion, this study establishes a proof-of-concept for using CCEVSe as a safe and effective biomimetic nanomedicine for targeted HCC therapy. By harnessing the body's own biological machinery, this approach offers a promising pathway toward the development of personalized, high-precision nanomedicines with minimized off-target toxicity and enhanced therapeutic efficacy.

## 5 Conclusion

In this study, we successfully engineered a biomimetic nanomedicine, CCEVSe, which utilizes tumor-derived extracellular vesicles to specifically target homologous tumor cells and deliver a payload of SeNPs. *In vitro* experiments demonstrated that CCEVSe exhibits superior homotypic targeting, effectively evades non-specific phagocytosis, and possesses enhanced tumor-killing ability. These findings were confirmed *in vivo*, where CCEVSe significantly inhibited tumor growth and achieved high selenium accumulation within tumors without causing damage to major organ tissues. Therefore, this safe and highly efficient CCEVSe platform represents a promising treatment strategy for a personalized and effective therapeutic approach using autologous tumor cells.

## CRediT authorship contribution statement

**Fang Cui:** Writing – original draft, Methodology, Investigation, Formal analysis, Data curation, Conceptualization. **Qi-xin Huang:** Methodology, Investigation, Formal analysis, Data curation, Conceptualization. **Zhuo Yu:** Methodology, Investigation, Formal analysis, Data curation, Conceptualization. **Jin-ju Yang:** Formal analysis, Data curation. **Shi-yu Liang:** Formal analysis, Data curation. **Mei-qi Wang:** Formal analysis, Data curation. **Jie Zhu:** Formal analysis, Data curation. **Chen Tian:** Formal analysis, Data curation. **Shao-xun Li:** Formal analysis, Data curation. **Hao-tian Wang:** Formal analysis, Data curation. **Fei Chen:** Formal analysis, Data curation. **Yang-jie Li:** Formal analysis, Data curation. **Xiaobin Feng:** Writing – review & editing, Project administration, Funding acquisition. **Rui-tian Liu:** Writing – review & editing, Project administration, Funding acquisition, Conceptualization. **Lingxiao Zhang:** Writing – review & editing, Project administration, Funding acquisition, Conceptualization.

## Declaration of competing interest

The authors declare the following financial interests/personal relationships which may be considered as potential competing interests:

Ruitian Liu reports financial support was provided by National Natural Scientific Foundation of China. Xiaobing Feng reports financial support was provided by National Natural Scientific Foundation of China. Lingxiao Zhang reports financial support was provided by European Union's Research and Innovation Program. Xiaobing Feng reports financial support was provided by Tsinghua University Initiative Scientific Research Program of Precision Medicine. If there are other authors, they declare that they have no known competing financial interests or personal relationships that could have appeared to influence the work reported in this paper.

## Data Availability

All data have been provided in the manuscript or Supplementary data.
