# Supplementary material for: Biomimetic selenium nanomedicine with homologous targeting enhances hepatocellular carcinoma therapy
Source: Mater Today Bio. 2025 Oct 21;35:102441. doi: 10.1016/j.mtbio.2025.102441 (PMC12593715; doi:10.1016/j.mtbio.2025.102441)
Supplement: Multimedia component 1 [file mmc1.docx]

**Supporting Information**

**Biomimetic selenium nanomedicine precisely targets tumor cells for enhanced hepatocellular carcinoma therapy**

*Fang Cui^1,2,3,†^, Qi-xin Huang^1,4,†^, Zhuo Yu^1,5,†^, Jin-ju Yang^1^ , Shi-yu Liang^1,3^, Mei-qi Wang^1,3^, Jie Zhu^1,3^, Chen Tian^1,3^, Shao-xun Li^1,3^, Hao-tian Wang ^1,3^, Fei Chen^1,6^, Yang-jie Li^1,6^, Xiaobin Feng^7,*^, Rui-tian Liu^1,*^, Lingxiao Zhang ^2,8,*^*

^1^ State Key Laboratory of Biopharmaceutical Preparation and Delivery, Institute of Process Engineering, Chinese Academy of Sciences, Beijing 100190, China

^2^ School of Medicine, Hangzhou City University, Hangzhou 310015, China

^3^ University of Chinese Academy of Sciences, Beijing 100049, China

^4^ Department of Neurology, Beijing Chaoyang Hospital, Capital Medical University, Beijing 100020, China

^5^ MD. Department of Medical Oncology, Beijing Tsinghua Changgung Hospital, School of Clinical Medicine, Tsinghua Medicine, Tsinghua University, Beijing 102218, China

^6^ Ningxia University, Yinchuan 750021, Ningxia, China

^7^ MD. Ytrrium-90 Precision Interventional Radiotherapy Center of Liver Cancer, Beijing Tsinghua Changgung Hospital, School of Clinical Medicine, Tsinghua Medicine, Tsinghua University, Beijing 102218, China

^8^ Interdisciplinary Nanoscience Center, Aarhus University, Aarhus C 8000, Denmark

^†^ These authors equally contributed to this work

***Corresponding Authors:**

Lingxiao Zhang, *E*-mail: [zhanglx@inano.au.dk](mailto:zhanglx@inano.au.dk)

Rui-tian Liu, *E*-mail: [rtliu@ipe.ac.cn](mailto:rtliu@ipe.ac.cn)

Xiaobin Feng, *E*-mail: [fxba02034@btch.edu.cn](mailto:fxba02034@btch.edu.cn)


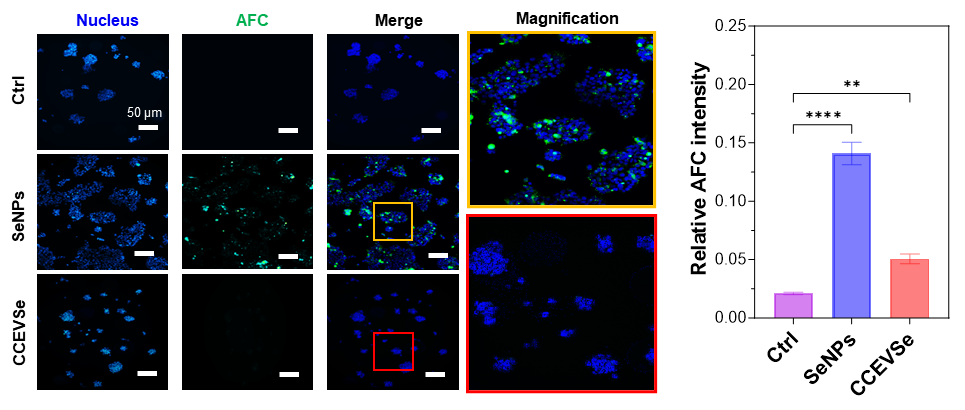


**Fig. S1 Cellular uptake of CCEVSe by HEK293 cells.** Cellular internalization of free SeNPs and CCEVSe was assessed in HEK293 cells after a 24-hour co-culture period. The cells were subsequently collected and imaged using confocal laser scanning microscopy. SeNPs were labeled with AFC (green fluorescence), and cell nuclei were stained with Hoechst 33342 (blue fluorescence). Data are presented as the mean ± SEM. The *p* values were analyzed by one-way analysis of variance, followed by multiple comparisons using Tukey’s test. **p* < 0.05, ***p* < 0.01, ****p* < 0.001, *****p* < 0.0001.

**Fig. S2** **Quantitative analysis of cellular uptake efficiency of CCEVSe by different cell lines.** The cellular uptake efficiency of SeNPs and CCEVSe at 10 μM mL^-1^ by HepG2-mCherry (A), HEK297 (B) and THP-1 (C) cells were analyzed at 24 h after incubation using flow cytometry. Data are presented as the mean ± SEM. The *p* values were analyzed by one-way analysis of variance, followed by multiple comparisons using Tukey’s test. **p* < 0.05, ***p* < 0.01, ****p* < 0.001, *****p* < 0.0001.


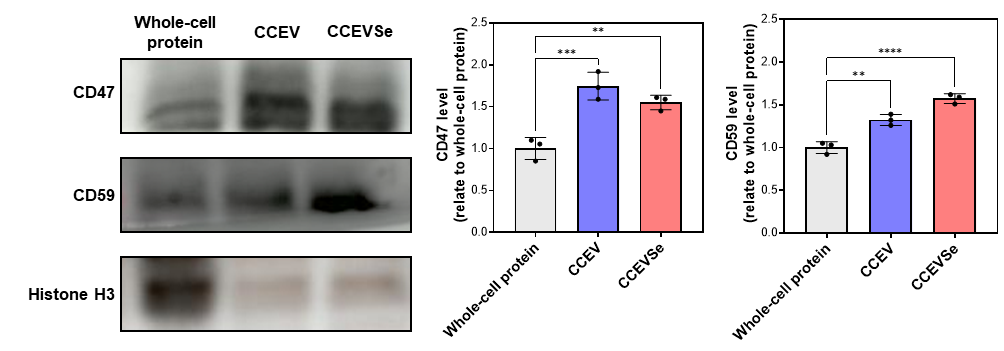


**Fig. S3 Expression of CD47 and CD59 in CCEVSe.** Western blot was used to detect the expression of CD47 and CD59 proteins in CCEV and CCEVSe. Then quantitative analysis of the band density was conducted. Data are presented as the mean ± SEM. The *p* values were analyzed by one-way analysis of variance, followed by multiple comparisons using Tukey’s test. **p* < 0.05, ***p* < 0.01, ****p* < 0.001, *****p* < 0.0001.

**Fig. S4 Cytotoxicity of CCEVSe.** The cytotoxicity of CCEV, SeNPs, CCEV&SeNPs (physical mixutre) and CCEVSe was evaluated on HepG2 cells. The drug concentration was determined using the basket-rotating method for concentration measurement. The concentration of CCEV was the same as that used in CCEVSe. Data are presented as the mean ± SEM. The *p* values were analyzed by one-way analysis of variance, followed by multiple comparisons using Tukey’s test. **p* < 0.05, ***p* < 0.01, ****p* < 0.001, *****p* < 0.0001.

**Fig. S5 Comparison of cytotoxicity of CCEVSe to first-line drugs.** The cytotoxicity of SeNPs and CCEVSe was evaluated on HepG2 cells by comparing with the clinical Sorarenib (A) and Doxorubicin (B). The drug concentration was determined using the basket-rotating method for concentration measurement. Data are presented as the mean ± SEM. The *p* values were analyzed by one-way analysis of variance, followed by multiple comparisons using Tukey’s test. **p* < 0.05, ***p* < 0.01, ****p* < 0.001, *****p* < 0.0001.

**
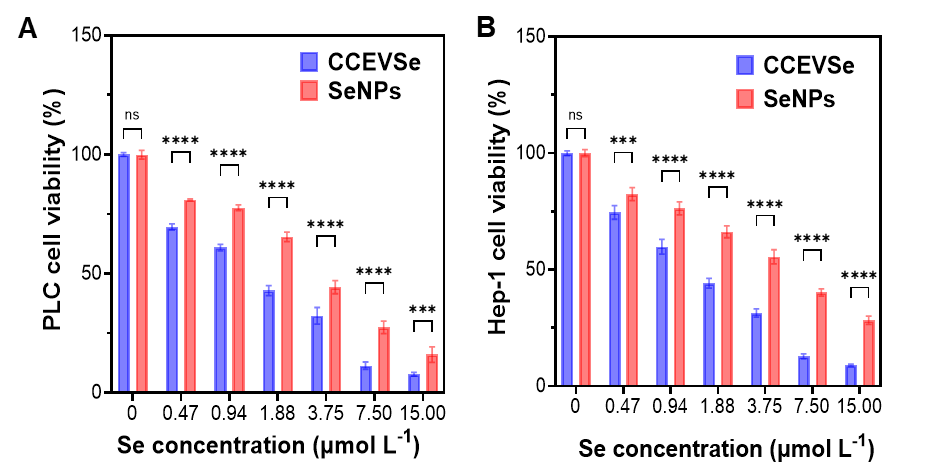
**

**Fig. S6 Cytotoxicity of CCEVSe in different HCC cell lines.** The cytotoxicity of SeNPs and CCEVSe was evaluated on PLC or Hep-1 cells using MTT assay. Data are presented as the mean ± SEM. The *p* values were analyzed by one-way analysis of variance, followed by multiple comparisons using Tukey’s test. **p* < 0.05, ***p* < 0.01, ****p* < 0.001, *****p* < 0.0001.

**
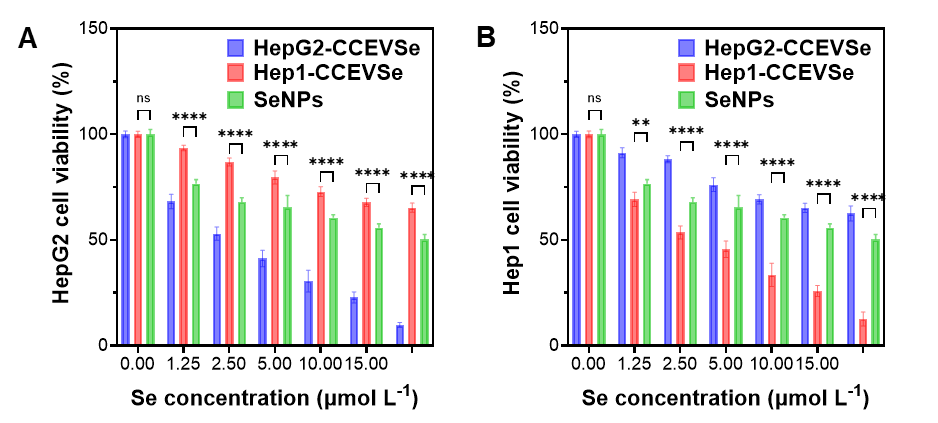
**

**Fig. S7** **Homologous targeting cytotoxicity of CCEVSe.** (A) Evaluation of the cytotoxicity of Hep1 cell-derived CCEVSe in HepG2 cells. (D) Evaluation of the cytotoxicity of HepG2 cell-derived CCEVSe in Hep1 cells. Data are presented as the mean ± SEM. The *p* values were analyzed by one-way analysis of variance, followed by multiple comparisons using Tukey’s test. **p* < 0.05, ***p* < 0.01, ****p* < 0.001, *****p* < 0.0001.

**
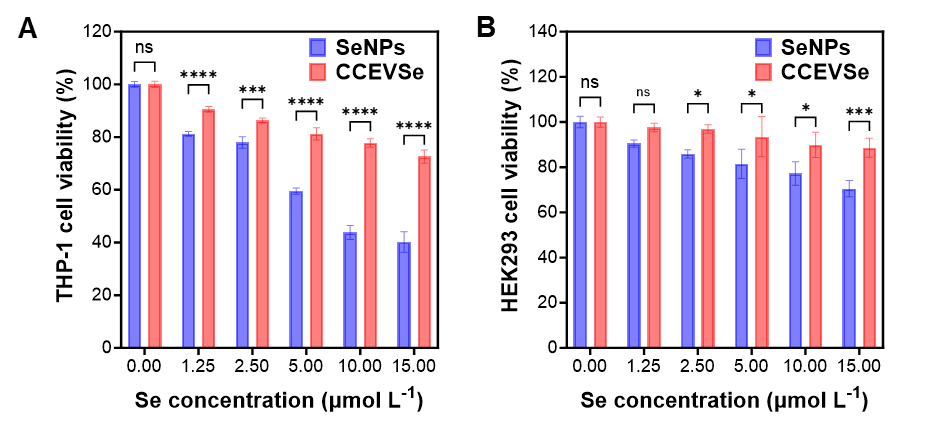
**

**Fig. S8 Cytotoxicity of CCEVSe to normal cell lines.** The cytotoxicity of SeNPs and CCEVSe was evaluated on THP-1 differentiated macrophages (A) and human embryonic kidney HEK293 cells (B) using MTT assay. Data are presented as the mean ± SEM. The *p* values were analyzed by one-way analysis of variance, followed by multiple comparisons using Tukey’s test. **p* < 0.05, ***p* < 0.01, ****p* < 0.001, *****p* < 0.0001.

**
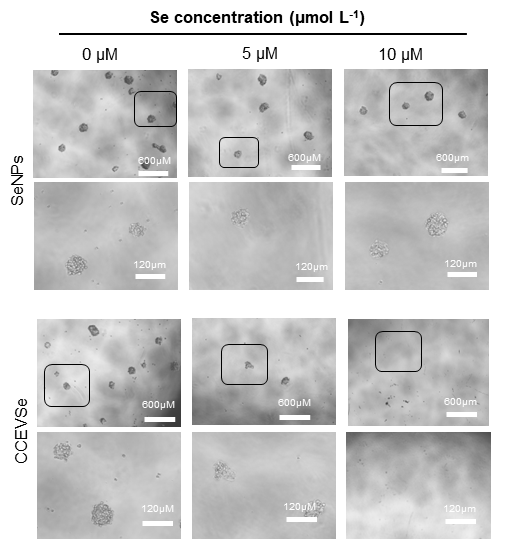
**

**Fig. S9 Optical images of tumor spheroid formation.** Tumor cells were cultured in ultra-low adhesion plates and treated with CCEVSe or SeNPs, and images were captured at different time points to monitor spheroid development.

**
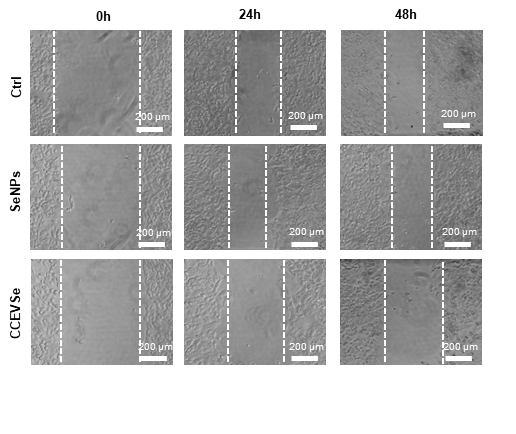
**

**Fig. S10 Inhibition of HepG2 cell migration by CCEVSe and SeNPs detected using a scratch assay.** Representative images show the migration of HepG2 cells after treatment, demonstrating that CCEVSe more effectively suppresses tumor cell migration compared with SeNPs.

**
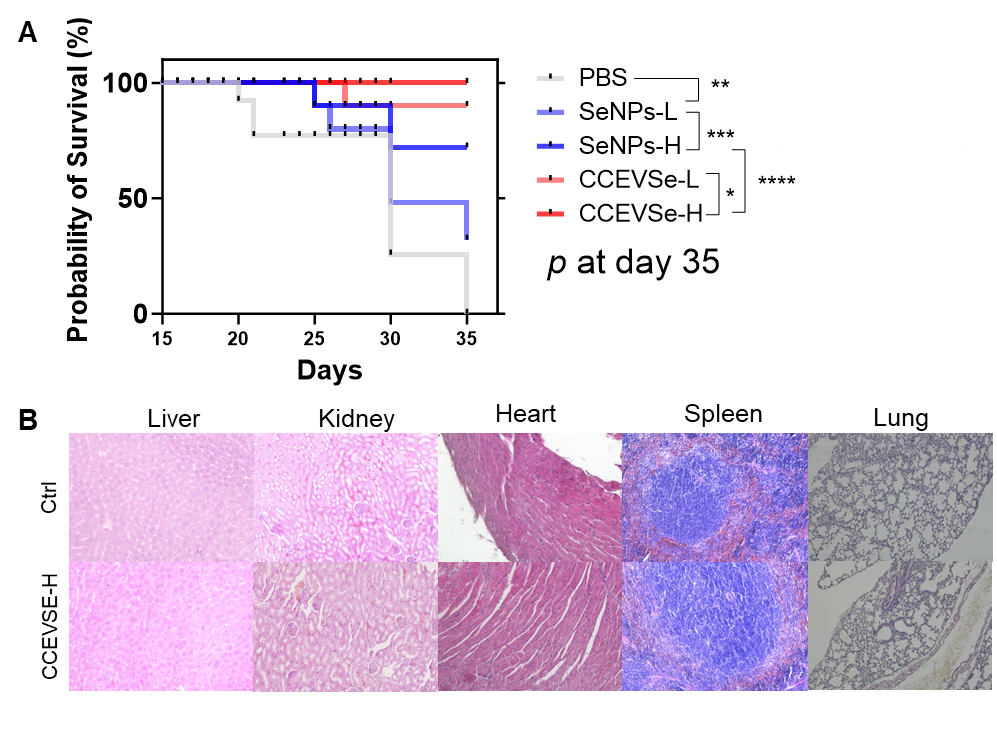
**

**Fig. S11 Evaluation of *in vivo* safety and survival.** (A) Survival of mice after different treatments. (B) H&E staining of various organs at the end of treatment in the high-dose CCEV-SeNPs group and the control group, showing no significant histological differences between the two groups. Data are presented as the mean ± SEM. The *p* values were analyzed by one-way analysis of variance, followed by multiple comparisons using Tukey’s test. **p* < 0.05, ***p* < 0.01, ****p* < 0.001, *****p* < 0.0001.

**Fig. S12 Detection of serum cytokines.** The levels of TNF-α and TFN-γ levels in mice were measured at 12 h after injection of SeNPs or CCEVSe. The changes in TNF-α and TFN-γ levels in mice were detected using an ELISA kit. Data are presented as the mean ± SEM. The *p* values were analyzed by one-way analysis of variance, followed by multiple comparisons using Tukey’s test. **p* < 0.05, ***p* < 0.01, ****p* < 0.001, *****p* < 0.0001.

**Fig. S13 Hemolysis risk of CCEVSe.** CCEVSe with varied Se concentrations (0.55-35 μmol mL^-1^) and Trion-X-100 with different concentration (w/v %) were added to fibrinogen-removed mouse blood cells to detect the percentage of red blood cell lysis.

**Table S1 DLS size, zeta potential and Se concentration of SeNPs and CCEVSe.**

|  | **Particle size (nm)** | **Zeta potential (mV)** | **Selenium concentration (mg L^-1^)** |
| --- | --- | --- | --- |
| **SeNPs** | 105 ± 3.6 | -52 ± 0.9 | 167.3 ± 0.86 |
| **CCEV** | 149 ± 1.5 | -15 ± 3.1 | / |
| **CCEVSe** | 150 ± 2.3 | -25 ± 1.6 | 35.27 ± 0.35 |

**Table S2 Dosage of the indicated formulations for animal treatment.**

|  | PBS | SeNPs-L | SeNPs-H | CCEVSe-L | CCEVSe-H |
| --- | --- | --- | --- | --- | --- |
| Injection volume | 200 μL | 200 μL | 200 μL | 200 μL | 200 μL |
| Injection dose  (measured by Se) | 0 μM | 12.5 μM | 25 μM | 12.5 μM | 25 μM |
